# QTL mapping under salt stress in rice using a Kalarata–Azucena population

**DOI:** 10.1007/s10681-022-03026-8

**Published:** 2022-05-15

**Authors:** Marjorie P. de Ocampo, Viet The Ho, Michael J. Thomson, Shiro Mitsuya, Akira Yamauchi, Abdelbagi M. Ismail

**Affiliations:** 1 International Rice Research Institute, DAPO Box 7777, Metro Manila, Philippines; 2 Graduate School of Bioagricultural Sciences, Nagoya University, Chikusa, Nagoya, 464-8601 Japan; 3 Faculty of Biology and Environment, Ho Chi Minh City University of Food Industry, Ho Chi Minh City, Vietnam; 4 Department of Soil and Crop Sciences, 343C Heep Center, Texas A&M University, College Station, TX USA

**Keywords:** Quantitative trait loci, Rice, Salt stress, Seedling stage, *Saltol*

## Abstract

Salt stress is a major constraint across large rice production areas in Asia, because of the high sensitivity of modern rice varieties. To identify quantitative trait loci (QTL) associated with salt tolerance in rice, we developed an F_2_ population from a cross between the salt-tolerant landrace, Kalarata, and the salt-sensitive parent, Azucena. F3 families from this population were screened and scored for salt tolerance using IRRI’s *Standard evaluation system* (SES). Growth, biomass, Na^+^ and K^+^ concentrations in leaf tissues, and chlorophyll concentration were determined. A genetic linkage map was constructed with 151 SSRs and *InDel* markers, which cover 1463 cM with an average distance of 9.69 cM between loci. A total of 13 QTL were identified using Composite Interval Mapping for 16 traits. Several novel QTL were identified in this study, the largest is for root sodium concentration (LOD = 11.0, R^2^ = 25.0) on chromosome 3, which also co-localize with a QTL for SES. Several QTL on the short arm of chromosome 1 coincide with the *Saltol* locus identified before. The novel QTL identified in this study constitute future targets for molecular breeding, to combine them with other QTL identified before, for higher tolerance and stable performance of rice varieties in salt affected soils.

**Supplementary Information:**

The online version contains supplementary material available at 10.1007/s10681-022-03026-8.

## Introduction

Rice is one of the most important cereal crops in the world. Among the common abiotic stresses affecting its productivity, salinity is a major constraint decreasing yields in both coastal and inland areas and both in irrigated and rainfed production ecosystems (Martinez-Atienza et al. 2007; Ismail et al. 2007; Ismail and Horie 2017). Salt stress is worsening in coastal areas because of salt intrusion due to sea level rise caused by climate change, and in inland areas because of the build-up of salinity as a consequence of excessive use of irrigation with improper drainage, and use of poor quality irrigation water (Ismail et al. 2010). The sensitivity of rice to salt stress depends on the growth and development stages; being relatively more tolerant at germination, becomes more sensitive at the early seedling and reproductive stages, and gains relatively greater tolerance during active tillering and grain filling (Ismail et al. 2007; Walia et al. 2007).

Salt tolerance at the seedling stage is attributed mainly to low Na^+^ accumulation and maintenance of low Na^+^:K^+^ ratio in the shoots (Lin et al. 2004; Platten et al. 2013). Therefore, Na^+^ exclusion mechanisms from shoots are crucial for survival in salt-affected lands (Ismail and Horie 2017). Quantitative trait loci (QTL) mapping has been frequently used to dissect and investigate major genes controlling salt tolerance and Na^+^ exclusion mechanisms from shoots of rice using various salt tolerant genotypes (Flowers et al. 2000; Koyama et al. 2001; Lin et al. 2004; Ming-Zhe et al. 2005; Lee et al. 2007; Mohammadi-Nejad et al. 2008; Sabouri and Sabouri 2008; Ammar et al. 2009; Pandit et al. 2010; Thomson et al. 2010b; Islam et al. 2011; Cheng et al. 2012; Ghomi et al. 2013; Mohammadi et al. 2013; Hossain et al. 2015; Tiwari et al. 2016; Ismail and Horie 2017). Among the several QTL mapped before, the availability of large effect QTL such as *Saltol* (Gregorio et al. 2002) provided opportunities to introduce these QTL into mega rice varieties or to combine them for multiple stress tolerance using marker assisted backcrossing (MABC) (Thomson et al. 2010a). The *Saltol* QTL, derived from the cross of IR29 and Pokkali, is flanked by markers RM23 and RM140 and has been mapped on chromosome 1, conferring salt tolerance at vegetative stage (Bonilla et al. 2002; Thomson et al. 2010b). This region extends from 9.8 to 12.2 Mb on the short arm of chromosome 1 (Walia et al. 2005) and accounts for about 45% of the phenotypic variance for Na^+^:K^+^ ratio in rice shoots at the seedling stage. Rice chromosome 1 harbors a hotspot of QTL for shoot Na^+^ content and Na^+^:K^+^ ratio (Negrao et al. 2011; Jing et al. 2017) where the *Saltol* QTL localizes, and related QTL have been reported from various salt tolerant rice landraces such as Nona Bokra (Lin et al. 2004), Pokkali (Thomson et al. 2010b; Alam et al. 2011), CSR 27 (Pandit et al. 2010), IR55178 (Koyama et al. 2001), Changbai10 (Zheng et al. 2015) and Co39 (Haq et al. 2008). On the other hand, various QTL for shoot Na^+^ content, Na^+^:K^+^ ratio and salt tolerance at the seedling stage have been reported on different chromosomes from various rice genotypes (Koyama et al. 2001; Haq et al. 2008; Thomson et al. 2010b), with novel QTL discovered from different donors. Therefore, mapping new QTL responsible for salt stress tolerance from new donors is necessary to combine them in high yielding backgrounds for high and stable salinity tolerance. Additional salt-tolerance-related QTLs from different donors will help rice breeders to have more choices in combining superior QTLs into one genetic background using gene pyramiding techniques, since the commonly used donor parents for salinity tolerance seem to possess few superior tolerance traits (Ismail et al. 2007). Combining genes for tolerance at all developmental stages will facilitate the development and release of new and more resilient rice varieties with substantially higher levels of salt tolerance (Thomson et al. 2010a).

Farmers in salt affected areas still grow traditional landraces despite their long duration, poor grain quality, and low yield because they possess moderate to high tolerance of salt stress (Ismail et al. 2007; Rahman et al. 2016). Several physiological mechanisms have been reported to be associated with salt tolerance in rice, including sodium exclusion, effective sequestration of toxic salts into older leaves and roots, and generation of antioxidants (Yeo and Flowers 1986; Ismail et al. 2007; Moradi and Ismail 2007; Rahman et al. 2016). Aside from characterizing the physiological responses to salt stress using salt tolerant landraces, advances have been made in identifying QTL and genes controlling salinity tolerance traits (Rahman et al. 2016). The present study aim to identify and map new major QTL associated with component traits involved in salt tolerance in rice, and to identify potential candidate genes for the promising QTLs using a new source of salt tolerance, Kalarata, and the salt-sensitive variety Azucena.

## Materials and methods

### Plant materials

A total of 400 F_2_ plants were developed from a cross between Kalarata and Azucena at the International Rice Research Institute (IRRI), Philippines. Kalarata is an *indica* landrace grown in the brackish-water paddy fields of Western India, and was recognized for its salt tolerance at the seedling, vegetative and reproductive stages, but not during germination (Pearson et al. 1966; Makihara et al. 1999; Rahman et al. 2016). At the seedling stage, the low Na^+^ accumulation in the shoots and high Na^+^ accumulation in roots of Kalarata suggests this genotype had developed salt exclusion mechanisms in roots to minimize salt transport to the shoot (Hedge and Joshi 1974; Rahman et al. 2016). Azucena, on the other hand, is a tall low tillering, long grain aromatic upland variety from the Philippines, of *tropical japonica* origin, and is sensitive of salt stress (Hittalmani et al. 2002).

F_2_ plants were advanced to constitute the phenotyping population (F_3_); then F_2_ plants were used for genotyping and F_3_families were phenotyped for salinity tolerance at seedling stage.

### Genotyping

#### DNA extraction

Two to three centimeter leaf samples collected from 14 day old greenhouse-grown F_2_ plants and their parents, Kalarata and Azucena, were used to extract genomic DNA using the DNA miniprep method. Frozen tissue was crushed and 800 µl of DNA extraction buffer (100 mM Tris–HCl, 50 mM EDTA, 500 mM NaCl, 1.25% (w/v) SDS, 3.8 g per L Sodium Bisulfite) was added to test tubes, and the tubes were vortexed and incubated at 65 °C for 20 min. Subsequently, a chloroform extraction was performed with 24:1 chloroform: isoamyl-alcohol solution, followed by an ethanol precipitation and resuspension in 100 µl of TE buffer. The quality and quantity of the isolated DNA were determined using a Spectrophotometer (Nanodrop, ND-1000, USA) and the DNA was diluted to working concentrations of 35 ng/μl with deionized distilled water.

A parental polymorphism survey covering the whole rice genome was performed using 257 SSR and insertion–deletion markers. Out of these markers, 151 were polymorphic. Each PCR reaction was carried out in a 15 μl volume mix containing 1.5 μl 10X PCR buffer, 1 μl of 1 mM dNTPs, 1 μl of 5 μM forward and reverse primers, 0.7 μl of 5 U/μl Taq polymerase, 8.8 μl deionized distilled water and 2 μl of DNA template at 35 ng/μl concentration. PCR profiles were programmed as follows: initial denaturation of 94 °C for 5 min, followed by 35 cycles of denaturation at 94 °C for 30 s, annealing at 55 °C for 30 s, extension at 72 °C for 45 s, and a final extension step at 72 °C for 5 min (MJ Research and G-storm thermal cyclers). PCR products were run on 6% (v/v) acrylamide gels at 100 V (Dual Triple-Wide Mini-Vertical System, C.B.S. Scientific, CA, USA) followed by SYBR-Safe staining (Invitrogen, USA), gel documentation (Alpha Innotech, USA), and manual scoring of the gel pictures.

### Evaluation of F_3_ families for salt tolerance

Due to a high degree of sterility, possibly due to the relatively high temperature inside the greenhouse, only 177 lines of the 400 F_2_ plants produced sufficient seeds; those 177 F_3_ families were evaluated for salt tolerance in a phytotron with day/night temperature of 29/21 °C and relative humidity of 70%. Seeds were heat treated for 5 d in a convection oven set at 50 °C to break their dormancy, then placed in petri dishes lined with moistened filter papers and incubated at 30 °C for 48 h to germinate. Pre-germinated seeds were sown on styrofoam floats (one seed per hole) with a net bottom floated on distilled water in 11 L plastic trays for 3 days, after which a nutrient solution (Yoshida et al. 1976) was used. At 14 d after seeding, salt stress was introduced by adding sodium chloride (NaCl) to an electrical conductivity (EC) of 6 dS m^−1^ for 3 days followed by EC of 12 dS m^−1^ until the experiment was terminated (total 21 days). The EC of the culture solution was monitored using an EC meter (HI9835, Hanna Instruments, Romania) and adjusted daily. Two replications were used, with ten individual plants per line evaluated for each replicate. IR29 (sensitive) and FL478 (highly tolerant) (Gregorio et al. 1997; Ismail et al. 2007) were used as checks. The pH of the nutrient solution was adjusted daily to 5.0, and the culture solution was replaced weekly.

### Assessment of physiological traits associated with salt tolerance

After treatment with NaCl at 12 dS m^−1^ for 21 days, entries were scored based on visual symptoms using IRRI’s Standard evaluation system (SES) scores, with ratings from 1 (highly tolerant) to 9 (highly sensitive (IRRI 2014). SES score of 1 indicates normal growth, only the old leaves show white tips while no symptoms on young leaves. SES score of 3 refers to near normal growth, with burning only in leaf tips, few older leaves become partially whitish. SES score of 5 indicates severely retarded growth with most old leaves severely injured and few young leaves elongating. SES score of 7, reflects complete cessation of growth, most leaves dried and only few young leaves still green. SES score of 9 when almost all plants are dead or dying.

The third fully expanded leaves were harvested and wrapped in aluminum foil and stored at − 15 °C. Root length (RL), shoot length (SL), root fresh weight (RFW) and shoot fresh weight (SFW) were recorded. Plants were harvested, washed thoroughly with deionized water, dried at 70 °C, and their root dry weight (RDW) and shoot dry weight (SDW) were determined. To assess Na^+^ and K^+^ concentrations in plant tissues, shoots and roots were extracted in 10 ml of 0.1 M acetic acid in a water bath set at 90 °C for 2 h, cooled at room temperature, and the evaporated solution replaced with deionized distilled water. Samples were then filtered and Na^+^ and K^+^ concentrations were measured using an atomic absorption spectrophotometer (AAS 3100, Perkin Elmer, USA). Respective root Na^+^:K^+^ ratio (RNKR) and shoot Na^+^:K^+^ ratio (SNKR) were calculated.

### Measurements of chlorophyll concentration in leaves

The third fully-expanded leaves were snap-frozen in liquid nitrogen and freeze dried, then ground to a fine powder and chlorophyll was extracted using 1.0 mg dry leaf material added to 1 mL of 80% (v/v) acetone. After extraction, the chlorophyll concentration was determined using an UV/VIS-Spectrophotometer (DU 530, Beckman Counter, USA). Readings were taken at 663, 652 and 645 nm wavelengths and the final chlorophyll concentration (ppm) was calculated using the following formulae (Arnon 1949):

$$\begin{aligned} {\text{Chlorophyll}}\;a & = 12.7_{{{\text{A}}663}} {-}2.7_{{{\text{A}}645}} \\ {\text{Chlorophyll}}\;b & = 22.9_{{{\text{A}}645}} {-}4.7_{{{\text{A}}663}} \\ {\text{Chlorophyll}}\;a\;{\text{and}}\;b & = 27.8_{{{\text{A}}652}} \\ \end{aligned}$$

### QTL analysis using Kalarata–Azucena population

QGENE software version 4.3.2 (Nelson 1997) was used to construct the genetic linkage map using Kosambi (1944) functions based on genotyping data of F_2_ plants. Marker orders were confirmed using the published physical map from the rice database (www.gramene.org) and Cornell map. The linkage groups were determined using command group with logarithm of odds (LOD) > 3.0; the same LOD was also used to check linkages among the SSR markers. The proportion of the total phenotypic variance explained by each QTL was calculated as R^2^ value (R^2^ = ratio of the sum of squares explained by the QTL to the total sum of squares). The analysis was carried out based on available information on genotype data from the genetic linkage map established for the Kalarata/Azucena cross. Composite interval mapping (CIM; Zeng 1994) was used to examine the association between phenotypic data and marker genotype. To increase the precision of putative QTL, minimal logarithm of odd (LOD) value was analyzed empirically from 1000 permutation tests (Churchill and Doerge 1994). This software was also used to identify the effects and origins of alleles contributed by the parents.

### Statistical analysis

Statistical analysis was performed for each trait based on a randomized complete block design model with two replications using R. Tukey’s Honestly least significance (HSD) was performed at the 0.05 significance level. Correlation analysis was performed in the mapping population to dissect physiological mechanisms that are associated with salt tolerance in this population at seedling stage. Correlations among traits were calculated using Spearman correlation method. Spearman’s correlation coefficient is a non-parametric measure of the strength and direction of association that exists between two variables measured on at least an ordinal scale. The test is used for either ordinal variables or for continuous data that has failed the assumptions necessary for conducting the Pearson’s product-moment correlation.

## Results

### Analysis of variance and correlation among physiological traits

Highly significant differences (*P* < 0.001) in SES scores, shoot and root dry weights, shoot and root lengths, shoot and root K^+^ and Na^+^ concentrations, and shoot and root Na^+^:K^+^ ratios were observed among families and parental lines. SES scores had the highest mean values (4.77) compared with other traits (Table 1). Differences in chlorophyll *a* and chlorophyll *a* and *b* among genotypes were also significant (*P* < 0.01). The highest values for the coefficient of variation (CV > 20%) were observed for root dry weight, shoot and root fresh weights, shoot Na^+^ concentration, chlorophyll *a*, chlorophyll *b*, and chlorophyll *a* and *b* (Table 1).

**Table 1:** Statistical parameters based on ANOVA analyses, among genotypes for growth and physiological traits in F_3_ families at seedling stage

Traits	Mean	CV (%)	MS error	Mean squares	*Pr* (> F)
SES scores	4.756	15.13	0.518	1.530	9.24e−14***
Shoot dry weight	0.185	19.34	0.001	0.004	< 2e−16***
Shoot length	48.20	9.956	23.03	74.34	1.65e−14***
Root dry weight	0.029	23.84	4.82e−05	1.23e−04	6.08e−10***
Root length	14.47	14.40	4.344	10.39	5.69e−09***
Shoot fresh weight	0.692	20.02	0.019	0.102	< 2e−16***
Root fresh weight	0.467	65.37	0.093	0.134	0.00731**
Shoot K^+^ concentration	2.120	2.379	0.003	0.234	< 2e−16***
Root K^+^ concentration	0.705	3.067	0.004	0.094	< 2e−16***
Root Na^+^ concentration	2.171	2.712	0.004	0.770	< 2e−16***
Shoot Na^+^ concentration	2.550	3.71	0.009	1.372	< 2e−16***
Shoot Na^+^:K^+^ ratio	1.261	4.710	0.004	0.506	< 2e−16***
Root Na^+^:K^+^ ratio	3.235	3.075	0.010	2.590	< 2e−16***
Chlorophyll *a*	0.437	53.14	0.054	0.084	0.00186**
Chlorophyll *b*	0.159	95.29	0.023	0.026	0.208
Chlorophyll *a* and *b*	0.606	51.66	0.098	0.153	0.00167**

Seedlings were subjected to salt stress of 12 dS m^−1^ for 21 days

Data are means of two replications; ** and *** indicates significant at *P* < 0.01 and 0.001, respectively using Tukey honestly significant difference (HSD)

The correlations among traits are presented in Table 2. Significant negative correlations were observed between SES and all other parameters related to salt tolerance such as shoot and root dry and fresh weights, shoot length, root K^+^ and Na^+^ concentrations, chlorophyll *a*, chlorophyll *b* and chlorophyll *a* and *b*. Shoot Na^+^ concentration correlated negatively with root Na^+^ concentration (r = − 0.16**) and with shoot K^+^ concentration (r = − 0.32**) (Table 2).

**Table 2:** Spearman correlation coefficients among different physiological traits in F_3_ families at seedling stage under salt stress of 12 dS m^−1^ imposed for 21 days

Trait	SES	Shoot dry weight	Shoot length	Root dry weight	Root length	Shoot fresh weight	Root fresh weight	Shoot K^+^ concentration	Root K^+^ concentration	Root Na^+^ concentration	Shoot Na^+^ concentration	Shoot Na^+^:K^+^ ratio	Root Na^+^:K^+^ ratio	Chlorophyll a	Chlorophyll b
Shoot dry weight	− 0.49^a^														
Shoot length	− 0.36^a^	0.77^a^													
Root dry weight	− 0.41^a^	0.84^a^	0.57^a^												
Root length	0.05^ns^	0.11^ns^	0.15^b^	0.16^b^											
Shoot fresh weight	− 0.49^a^	0.96^a^	0.74^a^	0.83^a^	0.10^ns^										
Root fresh weight	− 0.36^a^	0.79^a^	0.52^a^	0.89^a^	0.13^ns^	0.78^a^									
Shoot K^+^ concentration	0.09^ns^	− 0.11^ns^	− 0.16^ns^	− 0.04^ns^	− 0.16^ns^	0.03^ns^	− 0.12^ns^								
Root K^+^ concentration	− 0.33^a^	0.14^ns^	0.08^ns^	0.03^ns^	0.04^ns^	0.13^ns^	0.03^ns^	− 0.24^ns^							
Root Na^+^ concentration	− 0.27^a^	− 0.15^b^	− 0.12^ns^	− 0.04^ns^	− 0.03^ns^	− 0.11^ns^	− 0.04^ns^	0.14^ns^	− 0.07^ns^						
Shoot Na^+^ concentration	0.04^ns^	− 0.16^b^	− 0.09^ns^	− 0.16^b^	− 0.10^ns^	− 0.17^b^	− 0.11^ns^	− 0.32^a^	0.01^ns^	− 0.16^a^					
Shoot Na^+^:K^+^ ratio	0.10^ns^	0.12^ns^	0.13^ns^	0.07^ns^	0.04^ns^	0.14^ns^	0.09^ns^	0.39^a^	− 0.04^ns^	0.48^a^	− 0.44^a^				
Root Na^+^:K^+^ ratio	0.01^ns^	− 0.15^b^	− 0.12^ns^	0.18^b^	− 0.08^ns^	− 0.17^b^	− 0.14^ns^	− 0.32^a^	− 0.02^ns^	− 0.12^ns^	0.78^a^	− 0.33^a^			
Chlorophyll a	− 0.31^a^	0.44^a^	0.37^a^	0.34^a^	0.05^ns^	0.45^a^	0.26^a^	0.00^ns^	0.22^b^	− 0.08^ns^	− 0.18^b^	0.19^b^	− 0.16^b^		
Chlorophyll b	− 0.33^a^	0.40^a^	0.37^a^	0.32^a^	− 0.01^ns^	0.41^a^	0.25^a^	0.03^ns^	0.21^b^	− 0.03^ns^	− 0.17^b^	0.15^b^	− 0.15^b^	0.84^a^	
Chlorophyll a and b	− 0.32^a^	0.44^a^	0.39^a^	0.35^a^	0.04^ns^	0.46^a^	0.27^a^	0.00^ns^	0.22^b^	− 0.06^ns^	− 0.18^b^	0.19^b^	− 0.17^b^	0.98^a^	0.91^a^

*SES;* standard evaluation system score based on salt stress symptoms

^a^ and ^b^ indicate significance at *P* < 0.01 and 0.05, respectively, ns = not significant

### QTL identification

**Fig. 1:**
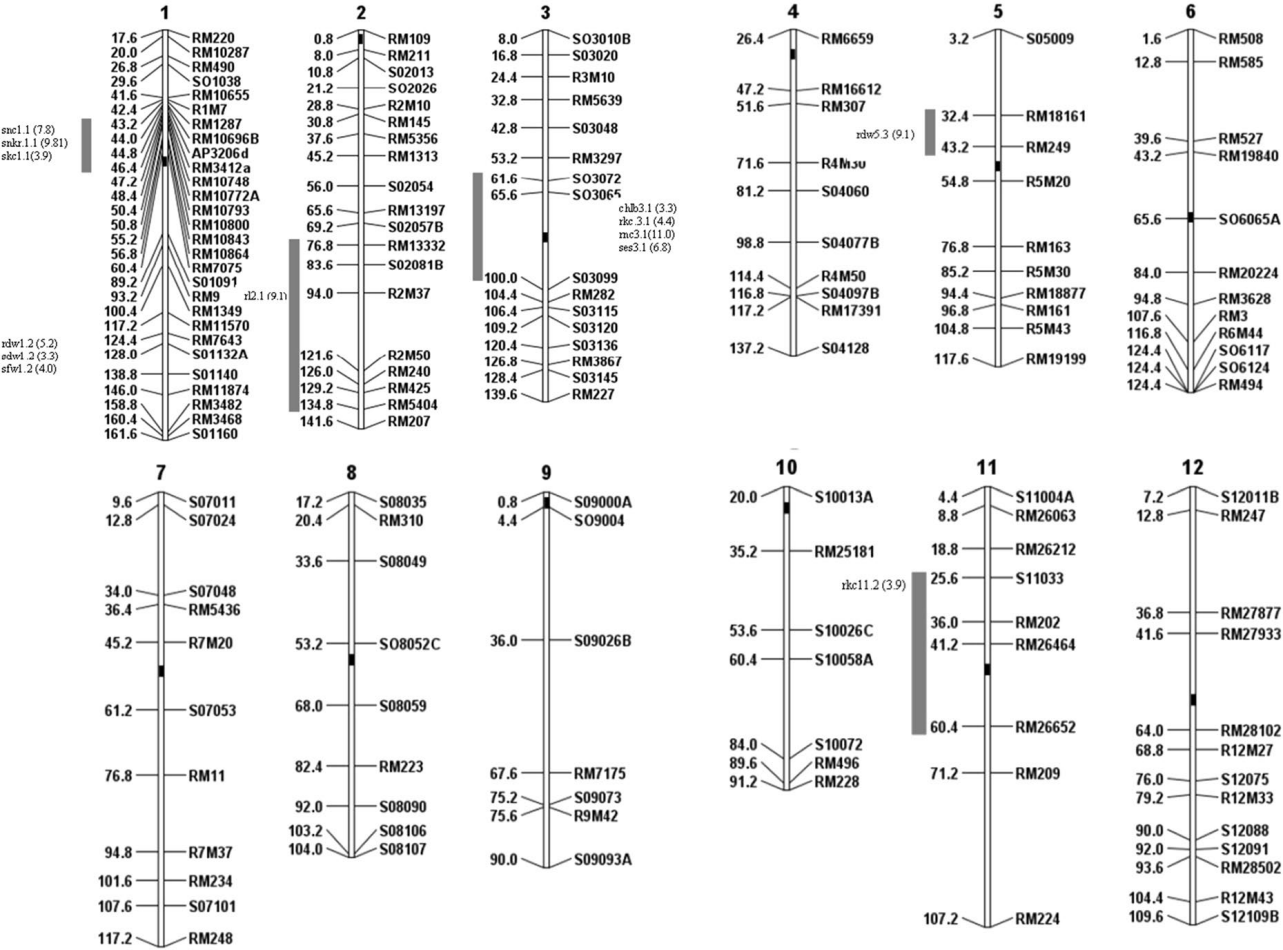
The genetic linkage map constructed with 151 markers across the 12 rice chromosomes, using the population derived from Kalarata/Azucena cross, with distances in cM converted from the physical map. The *LOD* scores are indicated in parenthesis and the labels on the right of the chromosomes reveal marker names, while the numbers on the left indicate marker positions. Black dots indicate centromere positions. Vertical bars on the left of each chromosome indicate the intervals of major QTL

Linkage analysis was performed with microsatellite genotyping data from the 151 SSR and *InDel* markers using QGENE version 4.3.2 (Nelson 1997). Figure 1 shows the distribution of the 151 markers throughout the rice genome, with a total length of 1463 cM. The average interval size between markers is 9.69 cM. QTL associated with salt tolerance were identified through CIM using QGENE program.

### QTL for physiological traits

**Fig. 2:**
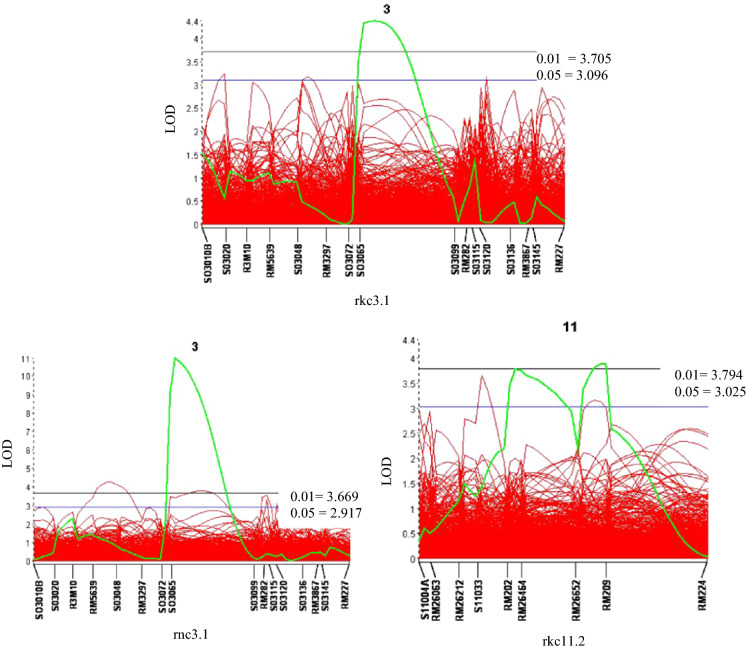
Significant QTL detected from Kalarata/Azucena population based on threshold LOD values at α_0.05_ (bottom line) and α_0.01_ (top line) after 1000 iterations in permutation analysis

Under salt stress, seven QTL for physiological traits with significant LOD value (3.3–11) were detected for chlorophyll *b* (*chlb3.1*), root K^+^ concentration (*rkc3.1*) and root Na^+^ concentration (*rnc3.1*) on chromosome 3, shoot K^+^ concentration (*skc1.1*), shoot Na^+^ concentration (*snc1.1*) and shoot Na^+^:K^+^ ratio (*snkr1.1*) on chromosome 1, and root K^+^ concentration (*rkc11.2*) on chromosome 11. These QTLs had R^2^ values of 8.3%, 11%, 25%, 9.6%, 19%, 23%, and 9.7%, respectively (Table 3; Figs. 1, 2).

**Table 3:** Significant QTL detected in F_3_ generation for traits related to salt tolerance from the cross of Kalarata/Azucena under salt stress condition at seedling stage based on composite interval mapping (CIM)

Traits	Chromosome	QTL name	Peak marker	Flanking markers	Peak LOD	Additive effect	Increased effect	R^2^ (%)	References
Agronomic traits Shoot fresh weight (SFW)	1	*sfw1.2*	RM7643	RM11570-SO1132A	4.0	0.095	A^a^	9.7	
Shoot dry weight (SDW)	1	*sdw1.2*	SO1132A	RM11570-SO1140	3.3	0.016	A	8.3	
Root dry weight (RDW)	1	*rdw1.2*	RM7643	RM11570-S01132A	5.2	0.0037	A	13.0	
	5	*rdw5.3*	RM18161	RM18161-RM249	3.2	0.0037	A	8.0	
Root length (RL)	2	*rl2.1*	R2M50	RM13332-RM5404	9.1	2.3	A	22.0	
SES	3	*ses3.1*	SO3065	SO3072-SO3099	6.8	0.71	A	17.01	
*Physiological traits*									
Shoot K^+^ concentration (SKC)	1	*skc1.1*	AP3206d	RM1287-RM3412a	3.9	− 0.1701	K	9.6	Koyama et al. (2001) (44.3–81.3 cM), Thomson et al. (2010a, b) (45.2–53.28 cM) and Negrao et al. (2011) (40.4–44.4 cM)
Root K^+^ concentration (RKC)	3	*rkc3.1*	SO3065	SO3072-SO3099	4.4	− 0.1	K	11.0	
	11	*rkc11.2*	RM26464	S11033-RM26652	3.9	0.079	A	9.7	
Shoot Na^+^ concentration (SNC)	1	*snc1.1*	RM10696B	RM1287-AP3206d	7.8	0.68	A	19.0	Koyama et al. (2001) (44.3–91.6 cM) and Thomson et al. (2010a) (43.2–50.4 cM)
Shoot Na^+^/K^+^ (SNKR)	1	*snkr1.1*	RM10696B	RM1287-AP3206d	9.81	0.48	A	23.0	Thomson et al. (2010a) (44.3–53.28 cM)
Chlorophyll b (CHLB)	3	*chlb3.1*	SO3065	SO3072-SO3099	3.3	− 0.42	K	8.3	
Root Na^+^ concentration (RNC)	3	*rnc3.1*	SO3065	SO3072-SO3099	11.0	− 0.74	K	25.0	

^a^K = Kalarata; A = Azucena; R^2^ = Phenotypic variance explained by a particular QTL; LOD = Logarithm of Odd. Additive effect: (Azucena–Kalarata)/2

### QTL for agronomic traits

Variation in growth of the seedlings was determined based on root length, root fresh and dry weights, shoot length, and shoot fresh and dry weights. Relevant QTL with significant LOD value (3.2–5.2) were detected for shoot fresh weight (*sfw1.2*), shoot dry weight (*sdw1.2*) on chromosome 1, root dry weight (*rdw1.2; rdw5.3*) on chromosome 1 and 5, and root length (*rl2.1*) on chromosome 2, with R^2^ values ranging from 8.3 to 22%. One QTL for SES scores was identified on chromosome 3 as *ses3.1*, with significant LOD value of 6.8 and R^2^ of 17.0% (Table 3; Fig. 1).

## Discussion

Salt tolerance in rice is a complex trait involving several physiological and adaptive mechanisms (Ismail et al. 2007; Ismail and Horie 2017). Agronomic and physiological traits seem to play crucial roles in salt tolerance. Significant differences between genotypes were observed for most of the growth and physiological parameters (Table 1 and Supplementary Table 1). SES scores based on visual salt-induced injury are often used for evaluating salt tolerance in rice at the seedling stage (Platten et al. 2013; Thu et al. 2017), with lower SES scores (1or 3) indicating higher tolerance. The significant negative correlations observed for SES score with shoot and root fresh and dry weights and shoot length demonstrate the significance and detrimental effects of high Na^+^ accumulation in plant tissues under salinity stress. Although salt tolerance evaluated by SES is attributed to low Na^+^ in shoots and high Na^+^ in roots in this study (Table 2), the mismatch of QTL for SES and for shoot Na^+^ concentration (Table 3) suggests the complexity of the physiological mechanisms associated with salt tolerance in Kalarata. Moreover, the diversity of QTL for SES among rice varieties also suggests that salt tolerance could be controlled by multiple mechanisms, multiple genes or alleles (Ismail and Horie 2017).

In this study, there was a negative correlation between SKC and SNC, suggesting potential competition between Na^+^ and K^+^ that occurred during uptake into the shoots resulting in a higher shoot Na^+^/K^+^ ratio. K^+^ is crucial for salt tolerance due to the fact that Na^+^ and K^+^ are physico-chemically similar (Lin et al. 2004; Sabouri et al. 2009). We identified 13 QTL for 5 traits of the shoot and 4 traits of the roots controlling growth and physiological attributes related to salt tolerance. Within the 13 QTL, ten of them were newly mapped in this study and the other three QTL for shoot K^+^ concentration, shoot Na^+^ concentration and shoot Na^+^:K^+^ ratio identified on chromosome 1 (Table 3; Figs. 1, 2) overlapped with QTL reported in previous studies. The QTL for shoot Na^+^ concentration and shoot Na^+^:K^+^ ratio were detected in the same position on the short arm of chromosome 1, which suggests that the loci affecting Na^+^ uptake also control Na^+^:K^+^ ratio in shoots, indicating potential functional relationships among these traits; or probability that either the same genes or tightly linked genes are involved in their control. The position of these QTL at 43.6 cM and 44 cM coincided with the well-known *Saltol* locus (Thomson et al. 2010b), and, respectively accounted for 19.0 and 23.0% of the phenotypic variation (Table 3). Similar results were reported in the study of Koyama et al. (2001) where they identified QTL controlling K^+^ concentration, Na^+^ uptake and Na^+^:K^+^ ratio in this region. Another QTL, *SKC1,* was also identified from a salt tolerant cultivar, Nona Bokra, on chromosome 1 at vegetative stage and found to encode a sodium transporter *HKT1;5* that helps control K^+^ homeostasis under salt stress (Lin et al. 2004; Ren et al. 2005). The identification of *OsHKT1;5* gene possess an additional exclusion mechanism in rice that functions as a low-affinity Na^+^ transporter and plays crucial roles in regulating the amount of Na^+^ transported from the root to the shoot (Platten et al. 2013; Rahman et al. 2016).

Three QTL for root K^+^ concentration (*rkc3.1* and *rkc11.2*) and root Na^+^ concentration (*rnc3.1*) identified on chromosomes 3 and 11 were new, providing tolerance through Na^+^ exclusion in the roots (Table 3; Fig. 2). Kalarata contributed the positive alleles of *rkc3.1* and *rnc3.1*. Also, *rnc3.1* QTL overlapped with QTL for SES (*ses3.1*), which agrees with a previous study (Krishnamurthy et al. 2011) showing that tolerant rice cultivars retain more Na^+^ in roots to limit Na^+^ entry into shoots. Kalarata alleles increased K^+^ concentration in both shoots and roots under salinity, indicating increased K^+^ uptake at the whole plant level by Kalarata alleles. Kalarata was reported to be tolerant at both vegetative and reproductive stages (Makihara et al. 1999). This data suggest that the salt tolerance in Kalarata at both seedling and reproductive stages is likely achieved by the overlapping QTLs for SES and root Na^+^ and K^+^ concentrations on chromosome 3, but not by the QTL on chromosome 1 that overlaps with *Saltol*, since *Saltol* QTL is reported to relate to tolerance at seedling stage but not at reproductive stage (Singh et al. 2021).

Azucena contributed to the positive alleles of *rkc11.2,* reflecting that alleles of QTL associated with salt tolerance were contributed by both parents. QTL for root Na^+^ concentration overlapped with root K^+^ concentration in the region SO3072-SO3099 on chromosome 3, which could be due to that salt tolerance is largely affected by ion exchange and homeostasis (Platten et al. 2013). The QTL detected for root Na^+^ and K^+^ concentrations were different from those detected for shoot traits. Lin et al. (2004) suggested that the genes controlling the transport of these two ions, Na^+^ and K^+^, between roots and shoots of rice seedlings might be different or are differentially regulated under salt stress. Koyama et al. (2001) also attributed the uptake of potassium to genes related to the structure or regulation of ion carriers and channels, while the transport of sodium under saline conditions is likely controlled by genes affecting root development, anatomy and architecture. Gregorio and Senadhira (1993) also observed two groups of genes involved in sodium and potassium uptake in rice; one group was envisaged to control sodium exclusion and the other to control potassium absorption. This could explain why there are different QTL for Na^+^ and K^+^ uptake in shoot and root. These findings are not only limited to rice, as Mano and Takeda (1997) reported that, salinity tolerance at seedling stage in barley is controlled by many minor genes and the expression of these genes seems to be affected by the environment. The study also suggested the importance of higher Na^+^ concentration in roots. Ismail and Horie (2017) pointed that responses to salt stress could be attributed to Na^+^ efflux from roots to the rhizosphere through salt overly sensitive (SOS1)– dependent Na^+^ exclusion, Na^+^ sequestration in vacuoles by tonoplast-localized Na^+^/H^+^ antiporters and Na^+^ loading and unloading at the xylem mediated by high-affinity K^+^ transporter (HKT) proteins. Hauser and Horie (2010) attributed prevention of Na^+^ accumulation in leaves to the combined action of transporters mediating Na^+^ unloading from xylem vessels and retaining it in roots to minimize accumulation to toxic concentrations in photosynthetic tissues, mediated by the high-affinity K^+^ transporter (HKT) proteins.

Tolerance at seedling stage seems to correlate poorly with tolerance at reproductive stage, suggesting different sets of traits are probably involved at each stage (Moradi et al. 2003). Despite the complexity, most salt-tolerant varieties seem to possess only few of the physiological mechanisms (sodium exclusion, effective sequestration of toxic salts into older tissues, stomatal responsiveness, higher tissue tolerance by compartmentation of salts into the apoplasts and upregulation of the antioxidant system during salt stress) indicating the prospects for developing highly tolerant rice varieties by combining superior alleles of genes controlling these traits (Ismail et al. 2007; Yeo and Flowers 1986; Moradi and Ismail 2007; Thomson et al. 2010b). Na^+^ exclusion from roots, sequestration of Na^+^ in roots, stems and basal portions of the leaf sheath, Na^+^ partitioning in older leaves and dilution of Na^+^ concentration in the large biomass of vigorous varieties are also proposed to influence leaf Na^+^ concentration (Platten et al. 2013). Several studies indicated the importance of Na^+^ execlusion from leaf blades as an important contributor to salt tolerance, with the leaf sheaths and roots acting as the main reservoirs for excess Na^+^ (Ren et al. 2005; Platten et al. 2013; Kobayashi et al. 2017; Neang et al. 2019).

Significant negative correlations were observed for SES score with the other traits (shoot length, shoot fresh and dry weights, root Na^+^ concentration, root K^+^ concentration, chlorophyll *a*, chlorophyll *b* and chlorophyll *a* and *b*) (Table 1). The QTL for SES, with Azucena as the source of the positive allele, overlapped with that for chlorophyll *b* (*chlb3.1*). A variety of QTL for visual salt-induced injury such as SES has been reported from different sets of rice crosses; for example, on chromosomes 1, 3, 4, 5 from a cross between CSR27 and MI-48 (Ammar et al. 2007), chromosomes 1, 3 from a cross between Milyang 23 and Gihobyeo (Lee et al. 2007), chromosomes 1, 4 from a cross between Hasawi and IR29 (Rahman et al. 2017), chromosomes 2, 4, 11 from GWAS study using 203 temperate japonica rice accessions (Batayeva et al. 2018), and chromosome 1, 3, 5, 12 from a cross between Capsule and BRRI dhan29 (Rahman et al. 2019). Three QTL controlling shoot fresh weight (*sfw1.2*), shoot dry weight (*sdw1.2*) and root dry weight (*rdw1.2*) were located in the same region in the long arm of chromosome 1, with Azucena as the source of positive alleles. These traits are related to seedling vigor, which is regarded as an important avoidance mechanism under salinity (Ismail et al. 2007; Reddy et al. 2017). The data indicates the important role of this region in determining biomass and vigor of rice. The QTL detected in this study provide a rich source of information for molecular breeding and for identifying useful genes for salt tolerance. Candidate genes associated with these QTL should further be assessed for their functional roles in physiological processes that confer salt tolerance in rice and for breeding improved varieties for salt affected areas. Haplotype and gene expression analysis will help extract more information on these candidate genes.

## Conclusions

The knowledge of QTL for salinity tolerance is an important step for future plant breeding programs to help increase and sustain yield of rice and other food crops grown in salt affected areas. In this study, SES scores showed strong association with shoot and root growth, shoot length, root K^+^ and Na^+^ concentrations, chlorophyll concentration, and shoot Na^+^ concentration. A total of 13 QTLs responsible for different traits likely associated with salinity tolerance were identified. QTL for SES (*ses3.1*) detected on chromosome 3 contributed by the positive alleles of Azucena and QTLs for root K^+^ concentration (*rkc3.1*) and root Na^+^ concentration (rnc3.1) detected on chromosome 3 contributed by the positive alleles of the salt tolerant Kalarata, are novel QTL associated with Na^+^ sequestration in roots. Identification of DNA markers that are closely linked with these new QTL will be useful for pyramiding multiple QTL, to develop varieties with greater salt tolerance for salt affected coastal and inland areas.

## Supplementary Information

Below is the link to the electronic supplementary material.

Supplementary file1 (DOCX 44 kb)

## Data Availability

The datasets supporting the conclusions of this article are included within the article and as supplementary files.
