# Supplementary material for: QTL mapping under salt stress in rice using a Kalarata–Azucena population
Source: Euphytica. 2022 May 15;218(6):74. doi: 10.1007/s10681-022-03026-8 (PMC9427886; doi:10.1007/s10681-022-03026-8)
Supplement: Supplementary file 1 — Supplementary file1 (DOCX 44 kb) [file 10681_2022_3026_MOESM1_ESM.docx]

**Supplementary Table S1. Greenhouse data for different agronomic and physiological traits among parents and genotypes in F3 families at seedling stage under salt stress of 12 dSm^-1^ for 21 days.**

|  |  |  |  |  |  |  |  |  |  |  |  |  |  |  |  |  |
| --- | --- | --- | --- | --- | --- | --- | --- | --- | --- | --- | --- | --- | --- | --- | --- | --- |
| Genotype | SES | SDW (g) | SL (cm) | RDW (g) | RL (cm) | SFW (g) | RFW (g) | SNC (%) | RNC (%) | SKC (%) | RKC (%) | SNKR | RNKR | CHLA (%) | CHLB (%) | CHLAB (%) |
| KA1 | 6 | 0.20 | 48.08 | 0.03 | 16.17 | 0.59 | 1.24 | 1.93 | 2.12 | 1.59 | 0.75 | 1.80 | 1.51 | 0.65 | 0.20 | 0.85 |
| KA2 | 5 | 0.13 | 40.75 | 0.03 | 13.83 | 0.44 | 1.90 | 2.49 | 2.17 | 1.50 | 1.00 | 2.37 | 1.87 | 0.05 | 0.02 | 0.08 |
| KA3 | 5 | 0.17 | 47.07 | 0.03 | 15.42 | 0.52 | 2.16 | 2.60 | 1.60 | 1.18 | 1.43 | 2.28 | 1.72 | 0.43 | 0.15 | 0.58 |
| KA4 | 5 | 0.15 | 44.53 | 0.03 | 15.83 | 0.52 | 1.42 | 2.02 | 2.02 | 1.54 | 0.73 | 2.04 | 1.59 | 0.55 | 0.17 | 0.72 |
| KA5 | 5 | 0.16 | 47.50 | 0.02 | 14.00 | 0.59 | 1.62 | 2.22 | 1.69 | 1.32 | 1.41 | 1.72 | 1.40 | 0.26 | 0.09 | 0.35 |
| KA6 | 5 | 0.16 | 47.33 | 0.03 | 13.75 | 0.64 | 1.78 | 2.29 | 1.78 | 1.41 | 1.04 | 2.17 | 1.62 | 0.42 | 0.13 | 0.56 |
| KA7 | 5 | 0.21 | 48.92 | 0.04 | 13.58 | 0.82 | 1.59 | 2.11 | 1.76 | 1.25 | 1.02 | 1.98 | 1.44 | 0.43 | 0.13 | 0.57 |
| KA8 | 4 | 0.20 | 55.17 | 0.03 | 12.33 | 0.74 | 1.74 | 2.47 | 1.77 | 1.22 | 1.16 | 2.29 | 1.61 | 0.29 | 0.09 | 0.39 |
| KA9 | 5 | 0.12 | 38.67 | 0.03 | 13.83 | 0.49 | 1.90 | 2.68 | 1.98 | 1.23 | 1.12 | 2.70 | 2.08 | 0.08 | 0.02 | 0.10 |
| KA10 | 5 | 0.25 | 54.83 | 0.04 | 12.30 | 1.35 | 1.90 | 2.12 | 2.16 | 1.30 | 0.81 | 2.30 | 1.91 | 0.19 | 0.06 | 0.26 |
| KA11 | 5.5 | 0.22 | 54.92 | 0.04 | 15.67 | 0.92 | 1.74 | 2.22 | 1.84 | 1.27 | 1.07 | 2.22 | 1.79 | 0.23 | 0.07 | 0.30 |
| KA12 | 5.5 | 0.17 | 47.42 | 0.03 | 14.83 | 0.71 | 1.55 | 2.28 | 1.80 | 1.21 | 1.05 | 2.22 | 1.65 | 0.43 | 0.14 | 0.58 |
| KA13 | 5.5 | 0.22 | 55.83 | 0.04 | 15.67 | 0.87 | 1.12 | 1.71 | 1.90 | 1.26 | 0.59 | 2.48 | 2.08 | 0.67 | 0.20 | 0.89 |
| KA14 | 5.5 | 0.24 | 64.17 | 0.04 | 16.67 | 0.90 | 1.15 | 1.88 | 2.03 | 1.19 | 0.62 | 2.99 | 2.59 | 0.44 | 0.14 | 0.59 |
| KA15 | 5.5 | 0.26 | 65.00 | 0.04 | 14.67 | 0.94 | 1.18 | 1.71 | 1.97 | 1.37 | 0.70 | 1.82 | 1.53 | 0.38 | 0.11 | 0.50 |
| KA16 | 6 | 0.18 | 49.17 | 0.03 | 21.00 | 0.46 | 1.65 | 2.29 | 1.96 | 1.38 | 0.92 | 2.17 | 1.70 | 0.81 | 0.17 | 1.06 |
| KA17 | 5 | 0.20 | 52.33 | 0.03 | 15.17 | 0.73 | 1.73 | 2.35 | 1.89 | 1.20 | 1.04 | 2.38 | 1.83 | 0.44 | 0.12 | 0.58 |
| KA18 | 6 | 0.16 | 41.33 | 0.03 | 15.17 | 0.62 | 1.66 | 2.40 | 1.89 | 1.28 | 1.15 | 2.17 | 1.71 | 0.18 | 0.05 | 0.24 |
| KA19 | 5 | 0.18 | 50.83 | 0.03 | 15.83 | 0.61 | 1.27 | 2.07 | 1.89 | 1.25 | 0.92 | 1.93 | 1.54 | 0.53 | 0.15 | 0.70 |
| KA20 | 5 | 0.19 | 52.33 | 0.03 | 15.17 | 0.61 | 1.01 | 1.81 | 1.90 | 1.17 | 0.64 | 2.64 | 2.35 | 0.33 | 0.09 | 0.43 |
| KA21 | 5 | 0.21 | 46.00 | 0.04 | 14.50 | 0.74 | 1.34 | 2.12 | 1.77 | 1.02 | 0.95 | 2.94 | 2.43 | 0.46 | 0.13 | 0.60 |
| KA22 | 5 | 0.18 | 54.17 | 0.03 | 16.08 | 0.64 | 2.17 | 2.77 | 1.71 | 1.07 | 1.57 | 2.87 | 2.12 | 0.23 | 0.06 | 0.30 |
| KA23 | 3 | 0.24 | 59.33 | 0.03 | 14.50 | 0.81 | 1.41 | 2.10 | 2.32 | 1.82 | 0.73 | 1.70 | 1.51 | 0.50 | 0.14 | 0.66 |
| KA24 | 5 | 0.20 | 44.08 | 0.04 | 18.67 | 0.78 | 1.13 | 1.78 | 2.19 | 1.62 | 0.64 | 1.77 | 1.55 | 0.53 | 0.15 | 0.71 |
| KA25 | 5 | 0.14 | 40.67 | 0.02 | 14.83 | 0.56 | 1.72 | 2.35 | 2.08 | 1.60 | 0.96 | 1.95 | 1.55 | 0.47 | 0.14 | 0.63 |
| KA26 | 5 | 0.13 | 42.83 | 0.02 | 10.50 | 0.50 | 1.93 | 2.75 | 2.07 | 1.56 | 1.04 | 2.10 | 1.57 | 0.31 | 0.08 | 0.40 |
| KA27 | 5 | 0.13 | 49.50 | 0.02 | 13.67 | 0.49 | 1.83 | 2.52 | 2.09 | 1.43 | 1.01 | 2.28 | 1.80 | 0.28 | 0.07 | 0.36 |
| KA28 | 5 | 0.18 | 48.50 | 0.03 | 13.83 | 0.71 | 1.18 | 1.86 | 2.20 | 1.61 | 0.60 | 2.17 | 1.72 | 0.40 | 0.12 | 0.54 |
| KA29 | 4 | 0.20 | 56.00 | 0.03 | 12.33 | 0.65 | 1.04 | 1.76 | 2.51 | 1.90 | 0.61 | 1.65 | 1.45 | 0.76 | 0.22 | 1.01 |
| KA30 | 5 | 0.16 | 54.33 | 0.03 | 11.83 | 0.62 | 1.65 | 2.51 | 2.32 | 1.77 | 0.94 | 1.86 | 1.48 | 0.27 | 0.08 | 0.36 |
| KA31 | 5 | 0.20 | 44.00 | 0.03 | 13.17 | 0.62 | 1.48 | 2.06 | 1.59 | 1.29 | 1.18 | 1.60 | 1.26 | 0.54 | 0.16 | 0.72 |
| KA32 | 5 | 0.19 | 40.83 | 0.03 | 16.08 | 0.64 | 2.15 | 2.71 | 1.89 | 1.38 | 1.26 | 2.21 | 1.62 | 0.26 | 0.08 | 0.35 |
| KA33 | 5 | 0.15 | 44.08 | 0.03 | 12.50 | 0.59 | 1.44 | 2.14 | 1.86 | 1.19 | 1.07 | 2.13 | 1.76 | 0.50 | 0.13 | 0.65 |
| KA34 | 4 | 0.15 | 39.33 | 0.03 | 12.50 | 0.51 | 1.29 | 1.76 | 2.09 | 1.59 | 0.72 | 1.55 | 1.34 | 0.29 | 0.08 | 0.38 |
| KA35 | 5 | 0.12 | 40.83 | 0.02 | 10.33 | 0.41 | 1.62 | 2.42 | 2.27 | 1.56 | 0.90 | 2.16 | 1.78 | 0.67 | 0.20 | 0.90 |
| KA36 | 5 | 0.13 | 40.75 | 0.02 | 12.58 | 0.66 | 1.48 | 2.25 | 2.21 | 1.55 | 0.91 | 1.85 | 1.59 | 0.35 | 0.11 | 0.48 |
| KA37 | 5 | 0.18 | 41.50 | 0.03 | 10.42 | 0.63 | 1.36 | 1.89 | 1.82 | 1.38 | 1.02 | 1.49 | 1.23 | 0.57 | 0.17 | 0.76 |
| KA38 | 5 | 0.16 | 44.08 | 0.03 | 9.92 | 0.65 | 1.76 | 2.45 | 1.81 | 1.24 | 1.25 | 2.13 | 1.55 | 0.07 | 0.00 | 0.08 |
| KA39 | 4.5 | 0.14 | 45.08 | 0.02 | 12.08 | 0.44 | 1.06 | 1.78 | 1.99 | 1.47 | 0.77 | 1.70 | 1.49 | 0.61 | 0.17 | 0.81 |
| KA40 | 5 | 0.14 | 41.83 | 0.03 | 12.25 | 0.51 | 1.13 | 1.65 | 1.91 | 1.36 | 0.69 | 1.86 | 1.71 | 0.46 | 0.13 | 0.61 |
| KA41 | 6 | 0.14 | 37.00 | 0.02 | 13.00 | 0.43 | 1.29 | 2.18 | 2.15 | 1.49 | 0.73 | 2.22 | 1.69 | 0.18 | 0.09 | 0.27 |
| KA42 | 4.5 | 0.22 | 38.83 | 0.04 | 13.08 | 0.90 | 0.80 | 1.50 | 2.04 | 1.35 | 0.56 | 1.88 | 1.65 | 0.45 | 0.17 | 0.64 |
| KA43 | 5 | 0.14 | 42.83 | 0.03 | 15.92 | 0.54 | 1.74 | 2.41 | 1.95 | 1.47 | 1.02 | 2.02 | 1.61 | 0.05 | 0.04 | 0.09 |
| KA44 | 5 | 0.15 | 48.50 | 0.02 | 13.00 | 0.56 | 1.36 | 2.15 | 2.21 | 1.52 | 0.66 | 2.44 | 2.23 | 0.21 | 0.13 | 0.35 |
| KA45 | 5 | 0.20 | 45.50 | 0.03 | 11.67 | 0.70 | 1.81 | 2.49 | 2.03 | 1.41 | 0.99 | 2.31 | 1.78 | 0.38 | 0.16 | 0.56 |
| KA46 | 5 | 0.16 | 43.33 | 0.03 | 12.17 | 0.60 | 1.55 | 2.35 | 2.07 | 1.61 | 0.89 | 1.91 | 1.40 | 0.40 | 0.16 | 0.56 |
| KA47 | 5 | 0.15 | 47.00 | 0.02 | 13.33 | 0.50 | 1.77 | 2.40 | 1.86 | 1.38 | 1.03 | 1.98 | 1.53 | 0.27 | 0.11 | 0.39 |
| KA48 | 5 | 0.19 | 48.83 | 0.03 | 10.55 | 0.65 | 1.31 | 1.97 | 2.14 | 1.66 | 0.82 | 1.56 | 1.30 | 0.39 | 0.13 | 0.53 |
| KA49 | 5 | 0.15 | 45.08 | 0.03 | 14.00 | 0.62 | 1.13 | 1.86 | 2.04 | 1.50 | 0.77 | 1.72 | 1.56 | 0.27 | 0.13 | 0.41 |
| KA50 | 4 | 0.21 | 46.17 | 0.03 | 12.67 | 0.78 | 1.08 | 1.73 | 2.04 | 1.44 | 0.72 | 1.66 | 1.56 | 0.43 | 0.17 | 0.62 |
| KA51 | 5 | 0.22 | 47.75 | 0.02 | 13.33 | 0.89 | 1.78 | 2.77 | 1.96 | 1.16 | 1.34 | 2.56 | 1.73 | 0.25 | 0.13 | 0.39 |
| KA52 | 5 | 0.22 | 52.08 | 0.03 | 13.33 | 0.77 | 1.34 | 2.08 | 2.07 | 1.56 | 0.83 | 1.75 | 1.41 | 0.57 | 0.21 | 0.79 |
| KA53 | 5 | 0.14 | 43.50 | 0.02 | 12.00 | 0.52 | 1.42 | 2.19 | 2.13 | 1.59 | 0.93 | 1.65 | 1.40 | 0.17 | 0.07 | 0.25 |
| KA54 | 5 | 0.20 | 44.25 | 0.03 | 11.67 | 0.70 | 2.02 | 2.67 | 2.24 | 1.63 | 1.00 | 2.09 | 1.60 | 0.97 | 0.32 | 1.32 |
| KA55 | 5 | 0.17 | 48.92 | 0.03 | 13.17 | 0.64 | 1.79 | 2.67 | 1.80 | 1.28 | 1.23 | 2.21 | 1.63 | 0.48 | 0.18 | 0.67 |
| KA56 | 5 | 0.16 | 43.25 | 0.02 | 11.83 | 0.55 | 1.76 | 2.39 | 1.55 | 1.19 | 1.21 | 2.09 | 1.50 | 0.36 | 0.15 | 0.52 |
| KA57 | 5 | 0.17 | 45.58 | 0.03 | 12.50 | 0.37 | 1.11 | 1.93 | 1.97 | 1.35 | 0.71 | 2.02 | 1.64 | 0.56 | 0.22 | 0.80 |
| KA58 | 5 | 0.14 | 39.92 | 0.02 | 12.75 | 0.45 | 1.58 | 2.16 | 1.70 | 1.24 | 1.10 | 1.94 | 1.46 | 0.32 | 0.13 | 0.47 |
| KA59 | 2.5 | 0.17 | 43.50 | 0.03 | 14.83 | 0.34 | 1.14 | 1.90 | 1.78 | 1.39 | 0.91 | 1.61 | 1.22 | 0.19 | 0.05 | 0.26 |
| KA60 | 5 | 0.15 | 44.08 | 0.03 | 12.42 | 0.46 | 1.66 | 2.32 | 1.79 | 1.17 | 1.20 | 2.24 | 1.68 | 0.44 | 0.14 | 0.59 |
| KA61 | 5 | 0.19 | 45.17 | 0.03 | 15.33 | 0.79 | 1.40 | 2.10 | 1.98 | 1.37 | 0.81 | 2.07 | 1.73 | 0.21 | 0.06 | 0.28 |
| KA62 | 5 | 0.17 | 44.33 | 0.03 | 14.00 | 0.67 | 1.54 | 2.49 | 1.97 | 1.22 | 1.18 | 2.29 | 1.66 | 0.53 | 0.18 | 0.72 |
| KA63 | 5 | 0.16 | 45.58 | 0.03 | 16.83 | 0.60 | 1.68 | 2.41 | 1.93 | 1.38 | 1.21 | 1.83 | 1.40 | 0.48 | 0.19 | 0.68 |
| KA64 | 5 | 0.23 | 59.33 | 0.03 | 15.50 | 0.88 | 1.27 | 1.76 | 1.84 | 1.43 | 0.80 | 1.62 | 1.27 | 0.79 | 0.23 | 1.06 |
| KA65 | 4.5 | 0.18 | 42.08 | 0.05 | 16.83 | 0.76 | 1.24 | 1.86 | 2.25 | 1.55 | 0.70 | 1.98 | 1.80 | 0.42 | 0.15 | 0.59 |
| KA66 | 6 | 0.13 | 45.00 | 0.02 | 16.00 | 0.36 | 1.71 | 2.55 | 1.91 | 1.36 | 1.02 | 2.30 | 1.77 | 0.04 | 0.01 | 0.05 |
| KA67 | 5 | 0.22 | 56.75 | 0.03 | 15.50 | 0.85 | 1.43 | 2.13 | 2.09 | 1.63 | 1.01 | 1.55 | 1.23 | 0.81 | 0.24 | 1.08 |
| KA68 | 5 | 0.18 | 47.17 | 0.03 | 20.00 | 0.59 | 1.26 | 2.03 | 2.17 | 1.54 | 0.80 | 1.93 | 1.73 | 0.38 | 0.15 | 0.55 |
| KA69 | 4.5 | 0.18 | 47.25 | 0.03 | 16.17 | 0.73 | 1.04 | 1.74 | 1.91 | 1.33 | 0.85 | 1.58 | 1.34 | 0.64 | 0.18 | 0.85 |
| KA70 | 5 | 0.17 | 43.25 | 0.03 | 16.92 | 0.65 | 1.24 | 1.99 | 1.63 | 0.64 | 0.67 | 2.64 | 1.22 | 0.18 | 0.06 | 0.24 |
| KA71 | 5 | 0.19 | 44.83 | 0.03 | 12.33 | 0.66 | 1.30 | 1.85 | 1.82 | 0.70 | 0.70 | 2.55 | 1.31 | 0.55 | 0.17 | 0.75 |
| KA72 | 6 | 0.19 | 57.00 | 0.03 | 18.33 | 2.40 | 2.46 | 1.28 | 2.10 | 1.55 | 0.51 | 1.44 | 1.33 | 0.82 | 0.23 | 1.09 |
| KA73 | 6 | 0.13 | 39.00 | 0.02 | 13.50 | 2.05 | 3.40 | 2.50 | 2.09 | 1.54 | 0.96 | 2.05 | 1.61 | 0.26 | 0.09 | 0.36 |
| KA74 | 6 | 0.11 | 37.50 | 0.03 | 18.00 | 2.10 | 3.76 | 3.01 | 1.89 | 1.34 | 1.30 | 2.51 | 1.69 | 0.29 | 0.09 | 0.39 |
| KA75 | 6 | 0.16 | 44.67 | 0.02 | 15.50 | 2.43 | 3.56 | 2.05 | 1.67 | 1.28 | 1.05 | 1.77 | 1.34 | 0.39 | 0.12 | 0.53 |
| KA76 | 6 | 0.14 | 43.50 | 0.02 | 13.00 | 2.21 | 3.19 | 2.30 | 1.78 | 1.23 | 1.12 | 2.15 | 1.63 | 0.42 | 0.13 | 0.57 |
| KA77 | 6 | 0.13 | 38.83 | 0.02 | 10.50 | 2.12 | 3.73 | 2.41 | 1.64 | 1.29 | 1.24 | 1.95 | 1.49 | 0.08 | 0.03 | 0.11 |
| KA78 | 6 | 0.12 | 40.83 | 0.02 | 16.00 | 1.81 | 3.53 | 2.82 | 1.83 | 1.35 | 1.32 | 2.16 | 1.59 | 0.33 | 0.09 | 0.44 |
| KA79 | 6.5 | 0.10 | 41.50 | 0.02 | 16.33 | 1.30 | 2.97 | 2.73 | 1.90 | 1.37 | 1.04 | 2.55 | 1.90 | 0.01 | 0.00 | 0.00 |
| KA80 | 6.5 | 0.18 | 42.17 | 0.04 | 17.00 | 3.54 | 4.23 | 1.91 | 2.13 | 1.53 | 0.62 | 2.28 | 1.87 | 0.16 | 0.04 | 0.21 |
| KA81 | 5 | 0.15 | 46.67 | 0.02 | 10.83 | 2.16 | 3.37 | 2.38 | 2.17 | 1.61 | 0.89 | 2.24 | 1.86 | 0.05 | 0.06 | 0.11 |
| KA82 | 5 | 0.15 | 44.67 | 0.02 | 12.17 | 2.46 | 3.70 | 2.29 | 2.04 | 1.42 | 1.02 | 2.06 | 1.75 | 0.14 | 1.15 | 0.41 |
| KA83 | 5 | 0.21 | 45.33 | 0.04 | 10.83 | 3.07 | 3.39 | 1.59 | 2.05 | 1.52 | 0.65 | 1.58 | 1.41 | 0.70 | 0.27 | 0.94 |
| KA84 | 5 | 0.16 | 45.50 | 0.04 | 14.33 | 3.03 | 3.81 | 2.19 | 2.16 | 1.39 | 0.89 | 1.99 | 1.65 | 0.11 | 0.04 | 0.15 |
| KA85 | 3.5 | 0.24 | 52.17 | 0.04 | 11.67 | 3.30 | 4.74 | 2.84 | 1.68 | 1.14 | 1.55 | 2.62 | 1.77 | 0.51 | 0.22 | 0.75 |
| KA86 | 3.5 | 0.22 | 56.67 | 0.03 | 12.67 | 2.99 | 3.58 | 2.03 | 1.98 | 1.29 | 0.79 | 2.37 | 2.00 | 0.71 | 0.27 | 1.00 |
| KA87 | 3.5 | 0.22 | 47.50 | 0.03 | 13.83 | 3.42 | 4.21 | 2.96 | 2.97 | 1.53 | 0.90 | 2.79 | 2.36 | 0.27 | 0.16 | 0.44 |
| KA88 | 3.5 | 0.20 | 48.50 | 0.03 | 16.00 | 2.93 | 3.85 | 2.24 | 1.78 | 1.37 | 1.26 | 1.71 | 1.35 | 0.39 | 0.19 | 0.59 |
| KA89 | 5 | 0.17 | 48.83 | 0.03 | 19.17 | 2.31 | 4.66 | 3.65 | 2.29 | 1.68 | 1.36 | 2.44 | 1.78 | 0.27 | 0.14 | 0.42 |
| KA90 | 5 | 0.24 | 55.17 | 0.04 | 14.83 | 2.60 | 2.67 | 1.54 | 2.10 | 1.51 | 0.60 | 1.60 | 1.40 | 0.33 | 0.13 | 0.47 |
| KA91 | 5 | 0.17 | 50.83 | 0.03 | 13.83 | 2.33 | 2.84 | 2.79 | 3.00 | 1.55 | 0.84 | 2.53 | 2.27 | 0.39 | 0.18 | 0.57 |
| KA92 | 5 | 0.22 | 48.67 | 0.03 | 17.00 | 2.65 | 3.39 | 2.96 | 2.58 | 1.42 | 1.13 | 2.45 | 1.88 | 0.64 | 0.24 | 0.91 |
| KA93 | 5 | 0.13 | 44.50 | 0.03 | 14.67 | 2.38 | 3.45 | 3.28 | 3.29 | 1.51 | 0.74 | 5.01 | 4.55 | 0.27 | 0.17 | 0.45 |
| KA94 | 5 | 0.17 | 50.00 | 0.03 | 14.83 | 2.24 | 2.66 | 1.09 | 1.45 | 1.49 | 0.69 | 0.71 | 0.86 | 0.53 | 0.30 | 0.84 |
| KA95 | 5 | 0.19 | 47.67 | 0.03 | 13.17 | 2.23 | 3.12 | 1.67 | 1.38 | 1.36 | 0.99 | 1.09 | 1.27 | 0.38 | 0.16 | 0.54 |
| KA96 | 5 | 0.14 | 52.67 | 0.02 | 13.50 | 1.94 | 4.97 | 5.46 | 3.10 | 1.62 | 2.00 | 3.64 | 2.43 | 0.34 | 0.11 | 0.47 |
| KA97 | 3.5 | 0.27 | 54.17 | 0.04 | 13.17 | 4.03 | 4.11 | 3.26 | 3.30 | 1.24 | 0.74 | 4.78 | 4.26 | 0.43 | 0.20 | 0.65 |
| KA98 | 3 | 0.17 | 49.33 | 0.03 | 12.83 | 2.44 | 3.00 | 2.90 | 2.95 | 1.54 | 0.83 | 2.87 | 2.42 | 0.22 | 0.14 | 0.36 |
| KA99 | 3 | 0.22 | 46.83 | 0.04 | 17.00 | 3.45 | 3.42 | 2.12 | 2.46 | 1.56 | 0.61 | 2.45 | 1.90 | 0.73 | 0.27 | 1.02 |
| KA100 | 5 | 0.15 | 48.67 | 0.02 | 13.33 | 1.83 | 2.62 | 2.81 | 2.38 | 1.26 | 1.00 | 2.87 | 2.22 | 0.32 | 0.09 | 0.42 |
| KA101 | 5 | 0.18 | 50.33 | 0.03 | 13.17 | 2.69 | 3.15 | 2.08 | 2.50 | 1.54 | 0.65 | 2.44 | 2.20 | 0.72 | 0.24 | 0.99 |
| KA102 | 5 | 0.18 | 48.50 | 0.03 | 13.33 | 2.64 | 3.48 | 2.76 | 2.47 | 1.31 | 1.00 | 2.70 | 2.18 | 0.54 | 0.20 | 0.76 |
| KA103 | 5 | 0.23 | 52.83 | 0.04 | 17.33 | 3.98 | 4.05 | 1.93 | 2.73 | 1.81 | 0.62 | 1.90 | 1.77 | 0.41 | 0.16 | 0.59 |
| KA104 | 5 | 0.18 | 47.50 | 0.04 | 13.33 | 3.19 | 3.88 | 2.81 | 2.66 | 1.46 | 0.99 | 2.57 | 2.10 | 0.34 | 0.30 | 0.81 |
| KA105 | 5 | 0.23 | 51.33 | 0.03 | 12.00 | 2.80 | 3.51 | 2.69 | 2.37 | 1.47 | 1.22 | 1.99 | 1.54 | 0.44 | 0.15 | 0.62 |
| KA106 | 5 | 0.12 | 41.33 | 0.02 | 14.00 | 2.08 | 3.46 | 3.03 | 2.37 | 1.26 | 1.40 | 2.65 | 1.99 | 0.26 | 0.08 | 0.36 |
| KA107 | 5 | 0.15 | 48.67 | 0.03 | 11.33 | 2.58 | 3.41 | 2.61 | 2.43 | 1.50 | 1.01 | 2.19 | 1.77 | 0.20 | 0.82 | 0.42 |
| KA108 | 5 | 0.16 | 44.50 | 0.02 | 14.33 | 2.75 | 3.91 | 2.69 | 2.13 | 1.20 | 1.10 | 2.86 | 2.27 | 0.13 | 0.07 | 0.20 |
| KA109 | 5 | 0.16 | 42.17 | 0.03 | 11.33 | 2.93 | 3.60 | 2.74 | 2.90 | 1.52 | 0.83 | 2.63 | 2.20 | 0.41 | 0.15 | 0.57 |
| KA110 | 5 | 0.23 | 56.33 | 0.04 | 14.83 | 3.50 | 3.92 | 2.42 | 2.46 | 1.44 | 0.80 | 2.59 | 2.20 | 0.35 | 0.11 | 0.48 |
| KA111 | 5 | 0.17 | 44.33 | 0.03 | 16.17 | 3.15 | 3.82 | 2.61 | 2.78 | 1.48 | 0.77 | 2.81 | 2.48 | 0.64 | 0.22 | 0.90 |
| KA112 | 6 | 0.13 | 42.33 | 0.02 | 15.50 | 2.36 | 3.50 | 2.58 | 2.32 | 2.28 | 1.13 | 1.13 | #DIV/0! | 0.18 | 0.08 | 0.26 |
| KA113 | 6 | 0.10 | 36.33 | 0.02 | 10.67 | 1.53 | 3.60 | 3.46 | 2.14 | 1.19 | 1.66 | 3.28 | 2.31 | 0.24 | 0.09 | 0.34 |
| KA114 | 6 | 0.15 | 50.17 | 0.03 | 12.17 | 2.48 | 3.49 | 3.04 | 2.67 | 1.41 | 0.94 | 3.26 | 2.67 | 0.26 | 0.08 | 0.35 |
| KA115 | 6 | 0.16 | 44.17 | 0.03 | 18.00 | 2.40 | 3.15 | 2.56 | 2.73 | 1.62 | 0.70 | 2.95 | 2.48 | 0.09 | 0.02 | 0.11 |
| KA116 | 6 | 0.13 | 43.00 | 0.02 | 16.00 | 2.19 | 3.17 | 2.27 | 2.07 | 1.24 | 0.98 | 2.36 | 1.85 | 0.60 | 0.20 | 0.83 |
| KA117 | 6 | 0.14 | 40.83 | 0.01 | 14.17 | 1.36 | 2.14 | 2.41 | 2.66 | 0.61 | 0.61 | 4.27 | 2.17 | 0.44 | 0.14 | 0.60 |
| KA118 | 4.5 | 0.21 | 56.33 | 0.04 | 16.33 | 3.66 | 4.11 | 2.51 | 2.36 | 1.42 | 0.99 | 2.13 | 1.72 | 0.54 | 0.17 | 0.73 |
| KA119 | 4.5 | 0.19 | 50.83 | 0.03 | 16.67 | 2.75 | 3.61 | 3.05 | 2.58 | 1.39 | 1.07 | 2.77 | 2.14 | 0.90 | 0.29 | 1.23 |
| KA120 | 4.5 | 0.19 | 48.33 | 0.03 | 12.67 | 2.49 | 3.11 | 2.59 | 2.74 | 1.58 | 0.81 | 2.48 | 2.10 | 0.72 | 0.23 | 0.98 |
| KA121 | 5 | 0.23 | 62.33 | 0.04 | 18.83 | 3.52 | 4.64 | 3.09 | 2.24 | 1.20 | 1.26 | 3.08 | 2.28 | 0.56 | 0.25 | 0.83 |
| KA122 | 5 | 0.27 | 60.67 | 0.04 | 16.50 | 3.95 | 3.98 | 2.23 | 2.53 | 1.34 | 0.68 | 2.67 | 2.41 | 0.28 | 0.14 | 0.43 |
| KA123 | 5 | 0.21 | 53.50 | 0.03 | 16.00 | 2.88 | 3.11 | 2.49 | 3.05 | 1.54 | 0.55 | 4.06 | 3.67 | 0.52 | 0.24 | 0.77 |
| KA124 | 5 | 0.16 | 45.83 | 0.02 | 14.67 | 2.33 | 3.10 | 2.71 | 2.82 | 1.36 | 0.74 | 3.61 | 3.36 | 0.34 | 0.13 | 0.48 |
| KA125 | 5 | 0.11 | 43.33 | 0.02 | 19.17 | 1.75 | 2.80 | 2.71 | 2.54 | 1.40 | 1.10 | 2.31 | 1.94 | 0.51 | 0.21 | 0.74 |
| KA126 | 5 | 0.20 | 54.83 | 0.03 | 19.67 | 2.80 | 4.11 | 2.91 | 1.94 | 1.18 | 1.47 | 2.73 | 2.01 | 0.37 | 0.18 | 1.03 |
| KA127 | 5 | 0.09 | 37.00 | 0.02 | 20.00 | 1.44 | 1.99 | 2.13 | 2.78 | 1.70 | 0.56 | 2.48 | 2.41 | 0.51 | 0.23 | 0.75 |
| KA128 | 5 | 0.15 | 49.83 | 0.02 | 13.67 | 1.91 | 3.02 | 2.84 | 2.19 | 1.11 | 1.10 | 3.48 | 2.72 | 0.43 | 0.19 | 0.63 |
| KA129 | 5 | 0.23 | 47.83 | 0.03 | 12.83 | 2.90 | 3.08 | 2.22 | 2.75 | 1.69 | 0.69 | 2.17 | 2.00 | 0.58 | 0.24 | 0.83 |
| KA130 | 3 | 0.26 | 55.00 | 0.04 | 13.33 | 3.54 | 3.85 | 2.07 | 2.42 | 1.53 | 0.65 | 2.35 | 2.02 | 0.56 | 0.20 | 0.78 |
| KA131 | 3 | 0.22 | 54.83 | 0.03 | 16.83 | 2.59 | 2.48 | 1.48 | 2.23 | 1.59 | 0.62 | 1.40 | 1.29 | 0.49 | 0.15 | 0.66 |
| KA132 | 3 | 0.22 | 54.50 | 0.04 | 16.00 | 3.68 | 4.03 | 1.85 | 1.92 | 1.31 | 0.87 | 1.74 | 1.47 | 0.52 | 0.17 | 0.69 |
| KA133 | 4 | 0.19 | 44.17 | 0.04 | 15.83 | 3.29 | 4.71 | 2.74 | 2.14 | 1.23 | 1.11 | 2.89 | 2.32 | 0.36 | 0.11 | 0.49 |
| KA134 | 4 | 0.25 | 57.33 | 0.04 | 15.50 | 3.67 | 4.43 | 2.60 | 2.05 | 1.15 | 1.03 | 2.95 | 2.27 | 0.71 | 0.27 | 1.03 |
| KA135 | 4 | 0.22 | 51.50 | 0.03 | 14.50 | 3.26 | 3.50 | 1.94 | 2.55 | 1.60 | 0.59 | 2.08 | 1.84 | 0.57 | 0.08 | 0.54 |
| KA136 | 4 | 0.20 | 54.50 | 0.02 | 15.00 | 2.10 | 3.25 | 2.74 | 2.23 | 1.40 | 1.12 | 2.22 | 1.75 | 0.49 | 0.18 | 0.69 |
| KA137 | 4 | 0.12 | 40.83 | 0.02 | 13.33 | 1.67 | 2.73 | 2.32 | 2.34 | 1.55 | 0.86 | 2.11 | 1.75 | 0.15 | 0.08 | 0.24 |
| KA138 | 4.5 | 0.18 | 48.33 | 0.02 | 18.33 | 2.14 | 3.66 | 2.92 | 1.70 | 1.22 | 1.57 | 2.41 | 1.62 | 0.33 | 0.09 | 0.43 |
| KA139 | 4 | 0.30 | 62.50 | 0.05 | 13.17 | 4.16 | 4.87 | 2.63 | 2.21 | 1.43 | 1.01 | 2.23 | 1.74 | 0.55 | 0.19 | 0.87 |
| KA140 | 3 | 0.27 | 62.83 | 0.04 | 13.17 | 3.32 | 3.59 | 2.11 | 2.49 | 1.68 | 0.69 | 2.12 | 1.77 | 0.71 | 0.22 | 0.96 |
| KA141 | 5 | 0.18 | 49.50 | 0.02 | 10.33 | 2.07 | 2.56 | 2.17 | 2.22 | 1.51 | 0.98 | 1.67 | 1.37 | 0.70 | 0.20 | 0.93 |
| KA142 | 5 | 0.12 | 34.17 | 0.02 | 15.33 | 2.00 | 2.91 | 2.52 | 2.43 | 1.79 | 0.90 | 1.91 | 1.56 | 0.48 | 0.16 | 0.67 |
| KA143 | 5 | 0.12 | 43.50 | 0.02 | 13.00 | 1.17 | 2.67 | 2.67 | 1.80 | 1.11 | 1.41 | 2.73 | 2.04 | 0.68 | 0.20 | 0.91 |
| KA144 | 4 | 0.26 | 57.83 | 0.04 | 15.83 | 3.15 | 3.88 | 2.62 | 2.33 | 1.41 | 1.00 | 2.31 | 1.88 | 0.84 | 0.26 | 1.13 |
| KA145 | 3 | 0.20 | 51.50 | 0.04 | 13.83 | 3.55 | 4.00 | 2.54 | 3.11 | 1.59 | 0.61 | 3.26 | 3.16 | 0.44 | 0.27 | 0.85 |
| KA146 | 3.5 | 0.27 | 55.33 | 0.04 | 10.50 | 3.09 | 3.58 | 2.19 | 1.99 | 1.22 | 0.81 | 2.95 | 2.58 | 0.83 | 0.27 | 1.13 |
| KA147 | 3 | 0.18 | 46.17 | 0.03 | 15.50 | 2.81 | 3.16 | 1.99 | 2.36 | 1.44 | 0.71 | 2.06 | 1.87 | 0.70 | 0.21 | 0.94 |
| KA148 | 3 | 0.17 | 45.67 | 0.03 | 11.67 | 2.24 | 3.15 | 2.51 | 2.31 | 1.44 | 1.02 | 2.15 | 1.67 | 0.74 | 0.23 | 0.99 |
| KA149 | 5 | 0.12 | 40.33 | 0.02 | 15.83 | 1.67 | 2.38 | 2.13 | 2.31 | 1.41 | 0.71 | 2.53 | 2.23 | 0.10 | 0.02 | 0.13 |
| KA150 | 5 | 0.19 | 49.83 | 0.03 | 12.83 | 2.85 | 4.44 | 3.15 | 2.21 | 1.44 | 1.09 | 3.02 | 2.28 | 0.33 | 0.10 | 0.45 |
| KA151 | 5 | 0.18 | 45.00 | 0.03 | 13.33 | 3.40 | 4.48 | 2.41 | 1.96 | 1.32 | 1.09 | 2.30 | 1.79 | 0.41 | 0.13 | 0.56 |
| KA152 | 5 | 0.10 | 39.67 | 0.02 | 12.17 | 1.57 | 3.04 | 2.71 | 1.94 | 1.23 | 1.32 | 2.46 | 1.85 | 0.15 | 0.04 | 0.20 |
| KA153 | 5 | 0.20 | 53.17 | 0.03 | 13.83 | 2.59 | 4.03 | 3.24 | 2.25 | 1.48 | 1.22 | 2.80 | 2.19 | 0.67 | 0.21 | 0.91 |
| KA154 | 5 | 0.18 | 52.00 | 0.02 | 18.67 | 1.96 | 2.36 | 2.48 | 2.76 | 1.64 | 0.83 | 2.23 | 1.97 | 0.60 | 0.19 | 0.82 |
| KA155 | 5 | 0.25 | 57.00 | 0.03 | 17.00 | 3.24 | 3.56 | 2.27 | 2.37 | 1.55 | 0.82 | 1.99 | 1.64 | 0.45 | 0.13 | 0.61 |
| KA156 | 5 | 0.13 | 40.00 | 0.02 | 18.67 | 1.82 | 2.26 | 1.78 | 2.32 | 1.58 | 0.72 | 1.68 | 1.58 | 0.43 | 0.11 | 0.56 |
| KA157 | 5 | 0.21 | 47.83 | 0.04 | 15.33 | 3.84 | 5.35 | 2.92 | 1.77 | 1.17 | 1.55 | 2.53 | 1.71 | 0.81 | 0.25 | 1.10 |
| KA158 | 5 | 0.27 | 52.00 | 0.04 | 14.50 | 4.06 | 4.15 | 1.86 | 2.10 | 1.21 | 0.61 | 2.88 | 2.56 | 0.60 | 0.20 | 0.82 |
| KA159 | 5 | 0.14 | 48.00 | 0.02 | 18.50 | 1.98 | 2.20 | 1.93 | 2.33 | 1.43 | 0.67 | 1.97 | 1.68 | 0.78 | 0.22 | 1.04 |
| KA160 | 4 | 0.27 | 52.83 | 0.04 | 15.00 | 3.12 | 3.89 | 2.71 | 2.18 | 1.31 | 0.99 | 3.01 | 2.37 | 0.24 | 0.07 | 0.33 |
| KA161 | 3.5 | 0.20 | 50.00 | 0.03 | 17.83 | 2.68 | 3.22 | 2.42 | 2.30 | 1.42 | 0.97 | 2.08 | 1.71 | 0.50 | 0.16 | 0.69 |
| KA162 | 3.5 | 0.26 | 57.33 | 0.04 | 16.17 | 3.92 | 5.01 | 2.81 | 2.13 | 1.28 | 1.28 | 2.61 | 2.06 | 0.52 | 0.21 | 0.75 |
| KA163 | 3.5 | 0.25 | 58.83 | 0.04 | 17.17 | 3.22 | 3.40 | 2.22 | 2.07 | 1.27 | 0.99 | 2.01 | 1.62 | 0.68 | 0.25 | 0.94 |
| KA164 | 3.5 | 0.17 | 40.67 | 0.03 | 15.33 | 3.05 | 4.02 | 2.57 | 2.18 | 1.43 | 1.33 | 1.88 | 1.52 | 0.34 | 0.12 | 0.47 |
| KA165 | 3.5 | 0.16 | 45.00 | 0.03 | 13.83 | 2.38 | 3.32 | 2.45 | 2.00 | 1.23 | 1.28 | 2.11 | 1.61 | 0.74 | 0.26 | 1.02 |
| KA166 | 3.5 | 0.20 | 48.67 | 0.03 | 16.17 | 2.88 | 3.56 | 2.33 | 1.99 | 1.30 | 1.06 | 2.10 | 1.61 | 0.13 | 0.05 | 0.18 |
| KA167 | 3.5 | 0.23 | 47.17 | 0.03 | 17.33 | 3.19 | 4.54 | 3.29 | 2.08 | 1.19 | 1.61 | 2.87 | 1.92 | 0.67 | 0.19 | 0.89 |
| KA168 | 4 | 0.22 | 47.83 | 0.03 | 17.83 | 2.71 | 4.13 | 3.35 | 1.91 | 1.20 | 1.82 | 2.80 | 1.81 | 0.25 | 0.07 | 0.34 |
| KA169 | 4 | 0.30 | 53.50 | 0.04 | 13.33 | 3.69 | 4.21 | 2.40 | 1.78 | 1.25 | 1.51 | 1.93 | 1.45 | 0.52 | 0.17 | 0.72 |
| KA170 | 4.5 | 0.33 | 52.67 | 0.05 | 11.83 | 4.30 | 4.74 | 2.37 | 2.18 | 1.44 | 1.05 | 1.93 | 1.58 | 0.56 | 0.16 | 0.75 |
| KA171 | 3.5 | 0.25 | 50.67 | 0.04 | 15.00 | 4.12 | 4.84 | 2.42 | 2.15 | 1.25 | 1.10 | 2.28 | 1.80 | 0.40 | 0.14 | 0.56 |
| KA172 | 4 | 0.25 | 50.67 | 0.04 | 12.33 | 3.60 | 4.43 | 2.67 | 1.97 | 1.16 | 1.25 | 2.68 | 2.06 | 0.63 | 0.19 | 0.85 |
| KA173 | 4.5 | 0.22 | 55.17 | 0.03 | 17.50 | 3.05 | 3.52 | 2.09 | 2.23 | 1.35 | 0.82 | 2.26 | 1.86 | 0.93 | 0.29 | 1.25 |
| KA174 | 4 | 0.22 | 49.50 | 0.03 | 12.17 | 2.98 | 3.75 | 2.57 | 1.54 | 1.20 | 1.25 | 0.83 | 0.00 | 0.64 | 0.20 | 0.87 |
| KA175 | 3.5 | 0.22 | 51.67 | 0.03 | 17.33 | 8.00 | 8.65 | 2.25 | 1.90 | 1.23 | 1.24 | 1.98 | 1.58 | 0.40 | 0.11 | 0.52 |
| KA176 | 3.5 | 0.27 | 53.00 | 0.04 | 14.67 | 3.66 | 4.08 | 2.09 | 1.90 | 1.24 | 1.04 | 1.98 | 1.60 | 0.35 | 0.10 | 0.47 |
| KA177 | 3.5 | 0.23 | 49.83 | 0.04 | 13.83 | 1.27 | 3.16 | 2.03 | 1.79 | 1.43 | 1.04 | 0.53 | 0.00 | 0.45 | 0.15 | 0.61 |
| Kalarata | 3 | 0.39 | 63.33 | 0.04 | 11.5 | 1.935 | 3.06 | 1.825 | 0.84 | 2.04 | 1.27 | 0.69 | 2.44 | 0.87 | 0.255 | 1.155 |
| Azucena | 9 | 0.165 | 46.165 | 0.03 | 14.83 | 0.755 | 1.925 | 1.645 | 0.93 | 2.48 | 2.825 | 1.715 | 2.66 | 0.225 | -0.05 | 0.065 |

SES: Standard Evaluation System scores; SDW: Shoot Dry Weight; SL: Shoot Length; RDW: Root Dry Weight; RL: Root Length; SFW: Shoot Fresh Weight; RFW: Root Fresh Weight; SNC: Shoot Na^+^ Concentration; RNC: Root Na^+^ Concentration; SKC: Shoot K^+^ Concentration; RKC: Root K^+^ Concentration; SNKR: Shoot Na^+^:K^+^ Ratio; RNKR: Root Na^+^:K^+^ Ratio; CHLA: Chlorophyll A; CHLB: Chlorophyll B; and CHLAB: Chlorophyll A and B
